# Deep Learning and Artificial Intelligence for the Determination of the Cervical Vertebra Maturation Degree from Lateral Radiography

**DOI:** 10.3390/e21121222

**Published:** 2019-12-14

**Authors:** Masrour Makaremi, Camille Lacaule, Ali Mohammad-Djafari

**Affiliations:** 1 Department of Orthodontics, University of Bordeaux, 33000 Bordeaux, France; masrour@makaremi.fr (M.M.); orthodontie@makaremi.fr (C.L.); 2 International Science Consulting and Training (ISCT), 91440 Bures-sur-Yvette, France

**Keywords:** Deep Learning (DL), Artificial Intelligence (AI), Convolutional Neural Network (CNN), classification, orthodontics, cervical vertebra maturation, machine learning

## Abstract

Deep Learning (DL) and Artificial Intelligence (AI) tools have shown great success in different areas of medical diagnostics. In this paper, we show another success in orthodontics. In orthodontics, the right treatment timing of many actions and operations is crucial because many environmental and genetic conditions may modify jaw growth. The stage of growth is related to the Cervical Vertebra Maturation (CVM) degree. Thus, determining the CVM to determine the suitable timing of the treatment is important. In orthodontics, lateral X-ray radiography is used to determine it. Many classical methods need knowledge and time to look and identify some features. Nowadays, ML and AI tools are used for many medical and biological diagnostic imaging. This paper reports on the development of a Deep Learning (DL) Convolutional Neural Network (CNN) method to determine (directly from images) the degree of maturation of CVM classified in six degrees. The results show the performances of the proposed method in different contexts with different number of images for training, evaluation and testing and different pre-processing of these images. The proposed model and method are validated by cross validation. The implemented software is almost ready for use by orthodontists.

## 1. Introduction and the Organisation of The Paper

Artificial Intelligence (AI), Machine Learning (ML) and Artificial Neural Networks (ANN) and many of their sub-fields such as Convolutional Neural Networks (CNN) and Deep Learning (DL) have became common tools in different areas of our life, and in particular, in biological and medical diagnostics. In this paper we show one more success in orthodontics.

In computer vision and particularly in image classification, deep convolutional neural networks (DCNNs) have emerged as the master algorithm and provided state-of-the-art performance in most tasks. The main power of a CNN is attributed to its deep architecture, allowing extracting a set of discriminating features at multiple levels of abstraction.

This paper is organized as follows: First, the importance of the work and its interest for the orthodontics community is mentioned. Then, the classical manual classification methods of radiographic images are described. The difficulties of the labeling task by the specialists are mentioned and the need for automatisation and the utility of ML tools are shown. Then, the proposed method is described which is mainly an appropriate CNN-based classification method which has been developed for these radiographic images.

## 2. Importance of the Work and Its Interest for the Orthodontics Community

Specialists in orthodontics are responsible for the treatment of dentofacial dysmorphisms, from different functional, genetical and morphological aetiologies. As a child or teenager is still growing, orthodontic treatment consists in a combination of orthodontics (about tooth position) and dentofacial orthopedics (about the guidance and stimulation of facial, maxilla and mandible growth in the three dimensions).

Many environmental and genetic conditions may induce upper or lower jaws’ lack of growth. Classically, to handle a treatment properly, every etiological condition that can be modified or corrected must be identified (diagnosis), normalized (treatment), and stabilized (retention). Specialists have to carefully examine and precisely analyze all the medical, functional, clinical and radiographic data in order to identify normal versus pathological conditions about tooth position, form or size, about lip, chin, cheeks, tongue and breathing functions, and about facial and jaws position and growing patterns. Adolescent orthodontic treatment also depends on proper management of jaws and facial growth to allow a balanced jaws position, maximize the airway and improve the facial appearance [1]. Treatment planning in orthodontics depends on a systematic diagnosis and prognosis.

Contemporary theories about craniofacial growth admit that the phenotype of the craniofacial complex is a result of a combination of genetic, epigenetic and environmental factors. The skeletal tissue of maxillomandibular complex grows due to sutures and osteogenic cartilages proliferation depending on genetic, intrinsic and extrinsic environment. So, facial growth can also be modified in amount and direction by extrinsic factors, including orthopedic and functional treatment. Thus, quantify facial and, in particular mandibular growth remaining, influences diagnosis, prognosis, treatment goals and planning. Indeed, apart choosing the good appliance needed to change the rate and direction of jaw growth, the right treatment timing is crucial. If high growth rate is about to occur, orthopedic treatment may permit to correct jaws, otherwise surgical correction of the jaw shift will be considered. The success of a dentofacial orthopedic treatment is linked to the determination of the interventional frame (periods of accelerated or intense growth) to maximize the chances to reach skeletal goals, with adapted methods and devices and in an optimized duration.

The most common dentofacial dysmorphism is the skeletal class II, corresponding to a short mandible. Study of normal mandibular growth and remodeling has shown different ways of bone formation that can be stimulated by functional and orthopedics treatments, in particular, condylar growth responsible for 80% of the mandible growth.

Numerous radiographic investigations have established that condylar/mandibular growth follows a similar growth curve as statural growth [2]. This growth pattern is characterized by variations of growth rate in four stages: first, a decrease of growth velocity from birth to 6 years old, then minor mid-growth spurt around 6–8 years, followed by a pre-pubertal plateau with decelerated growth rate, and finally, the facial growth curve describes a peak of growth velocity corresponding to the pubertal growth spurt, which coincides, precedes or follows (from 6 to 12 months) the statural growth peak (controversial) [3]. This spurt occurs approximately two years earlier in some girls than in some boys [4].

To estimate mandibular growth potential, the patient must be localized on is growth curve, and many biologic indicators have been proposed; increase in body height, menarche, breast and voice changes, dental development and eruption, middle phalanx maturation of the third finger, maturation of the wrist and cervical vertebral maturation [5,6,7,8].

## 3. The Classical Radiographic Manual Methods

### 3.1. Hand-Wrist Radiograph Method HWM:

The comparison method describes in the Atlas of Greulich et Pyle in 1959 or the Fishman’s method in 1982, permits to identify specific ossification stages occurring before, during, or after mandibular growth peak, on left hand and wrist radiographs [9,10]. Hand wrist radiographs have been used as a gold standard in the assessment of skeletal maturation for many decades, but have presented several issues such as additional x-ray exposure, time and experience required (even if a digital software is now available [11]), and a sexual dimorphism and ethnic polymorphism in morphological modifications [12,13]

### 3.2. Vertebrae Maturation CVM:

The first to propose to predict skeletal age and growth potential by cervical vertebrae maturation (CVM) method is LAMPARSKI in 1972. Cervical vertebrae are available on the lateral cephalometric radiographs, prescribed routinely by orthodontists for each patient diagnosis and treatment planning [14]. He used measurements of mandibular length on several annual lateral cephalograms to describe individual mandibular growth curve, and correlated it with morphological description of vertebrae morphology at each stage. This method was modified several times first by Hassel and Farman (1995) [15], then twice by Baccetti et al. (2002 and 2005) for a more accurate assessment of cervical maturation, by six stages identified by morphological changes in the C2,C3,C4 vertebral bodies on a single lateral cephalogram, independently of patient gender [16].

This last version is the most used to detect the mandibular growth spurt, as it shows the best results in clinical applicability [17].

**Figure 1:**
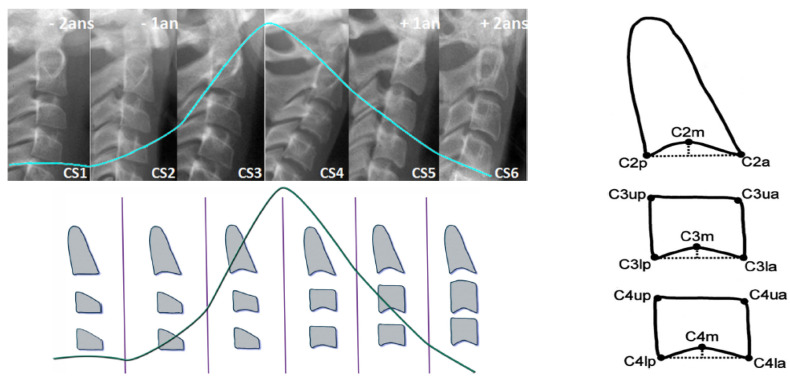
(**Left**) CVM radiological and morphological stages superposed with Björk growth curve [16]; (**Right**) Cephalometric landmarks for CVM stages determination [1].

As most bones of the human body, vertebrae growth and maturation change from birth to full maturity. Cervical vertebrae are the first seven pieces of the spinal column. Vertebral growth in the cartilaginous layer of the superior and inferior surfaces of each vertebrae involves changes in size of vertebral bodies and shape of upper and lower borders of C2,C3,C4 vertebrae. These changes have been described into six stages, correlating with morphological modifications of the vertebral shapes and estimated time lapse from the mandibular growth peak. Both visual and cephalometric appraisals of morphological changes have been proposed. See Figure 1.

Visual analysis [1]:

- Cervical stage 1 (CS1) = 2 years before mandibular growth peak:
  Lower borders of C2 to C4 vertebrae are flat. C3 and C4 superior borders are tapered from posterior to anterior.
- Cervical stage 2 (CS2) = 1 year before mandibular growth peak:
  Lower border of C2 presents a concavity. Bodies of C3 and C4 are the same.
- Cervical stage 3 (CS3) = during the year of the mandibular growth peak:
  Lower borders of C2 and C3 present concavities. Vertebrae are growing so C3 and C4 may be either trapezoid or rectangular shape, as superior borders are less and less tapered.
- Cervical stage 4 (CS4) = 1 or 2 years after mandibular growth peak:
  Lower borders of C2, C3 and C4 present concavities. Both C3 and C4 bodies are rectangular with horizontal superior borders longer than higher.
- Cervical stage 5 (CS5) = 1 year after the end of mandibular growth peak:
  Still concavities of lower borders of C2, C3 and C4. At least one of C3 or C4 bodies are squared and spaces between bodies are reduced.
- Cervical stage 6 (CS6) = 2 years after the end of mandibular growth peak:
  The concavities of lower borders of C2 to C4 have deepened. C3 and C4 bodies are both square or rectangular vertical in shape (bodies higher than wide)

#### Cephalometric Appraisals:

Using the landmarks illustrated on Figure 1(right), cephalometric analysis consists in the measurement of:

- The concavity depth of the lower vertebral border (estimated by the distance of the middle point (Cm) from the line connecting posterior to anterior points (Clp-Cla))
- The tapering of upper border of vertebral C3 and C4 bodies (estimated by the ratio between posterior and anterior bodies heights (Cup-Clp)/(Cua-Cla))
- The lengthening of vertebral bodies (estimated by the ratio between the bases length and anterior bodies borders height (Clp-Cla)/Cua-Cla)

Many researchers found this method as valid and reliable as hand and wrist Xray [14]. The cervical vertebrae maturation stages have been demonstrated as a clinically useful maturation indicators for evaluation of pubertal growth height and mandibular velocities [18,19,20], by correlation between chronological age and cervical vertebrae maturation, between hand–wrist and cervical-vertebrae maturation [16,21,22,23].

Some studies underlined the need for association with other clinical assessments [24] in clinical practice, and a good reliability in differentiating pre and post mandibular growth spurt periods [25].

### 3.3. The Difficulties of the Labeling Task

Specific training is provided to assess CVM stages reliably, and repeatably at a satisfactory level [26,27]. Gabriel et al. minimized the risk of bias (radiographs without tracings, standardized training to private practice orthodontists...) and observed a moderate intra and inter-observer agreement (30%–62% of cases). These results confirm the expertise required to properly determine the CVM stage, and may be explained by the use of a qualitative method of assessment, and the lack in detecting exceptional cases (individual variations in size and morphology, outside the norms defined by the method). Moreover, for orthodontists, the cervical vertebrae area on the lateral cephalograms is outside their expertise of “visual fields”. They have poor general knowledge and experiences about vertebrae observation, as they focus on maxillomandibular bones and teeth at a first glance. This would have been a difficulty in the labeling task of our radiographs.

In our work, all lateral radiographs have been labeled by a radiological technician, specialized in cephalometric tracing and over trained in CVM stages agreement (3 years full time), using a standardized morphological and cephalometrical protocol. Intra observer reproducibility must be estimated in further study.

### 3.4. The Need for Automatisation and the Help Which It Brings

Estimation of CVM stage represents only one single element influencing the patient orthodontic treatment. The practitioner must master the entire clinical, functional, bio-mechanical and cephalometric data analysis in order to define proper diagnosis and treatment goals and planning. Even in being specialists, orthodontists require a very broad range of skills and a great deal of time for each patient to complete diagnosis. Considering that reproducibility of classifying CVM stages is superior at 98% by trained examiners [1], automatisation by expert eyes will provide time saving, efficiency, accuracy, repeatability in treatment planning and patient care.

Few studies have presented software programs for cephalometric determination of C2, C3 and C4 vertebrae proportions according reference points marked manually on the image, and automatically calculates the skeletal maturation stage. This computer-assisted analysis still depends on operator experience [28]. Padalino et al. ran a study comparing manual analysis of CVM stages and the analysis performed by a dedicated software. It has shown a concordance of 94% between the two methods but hand-tracing analysis was quicker of 28 s on average [29].

Deep Learning (DL) Convolutional Neural Networks (CNN) have already been used to diagnose metabolic disorders in pediatric endocrinology, in order to assess skeletal bone age on left hand-wrist radiographs. Deep learning approach proposes better accuracy than conventional methods in processing the image in less than 1 s. Our study aims to develop a fully automated deep learning assessment of CVM stages on lateral cephalograms in orthodontics.

## 4. Proposed Method

The proposed method is mainly a supervised classification method using CNN and DL. The main contributions of this paper are:

- Appropriate needed preprocessing of the images before starting the training, evaluation and testing steps;
- A Comparison of different appropriate CNN and DL existing methods on a reduced set of images in our data base;
- Appropriate choice of a DL CNN structure, criteria, optimization algorithm and setting of all the parameters and hyper parameters for our application;
- Showing the performances of the proposed method for a different number of training, evaluating and testing images;
- Pre-clinical evaluation of the implemented method.

These contributions are presented in the following subsections.

### 4.1. Preprocessing of the Data

For this classification task, we had an image data base of 2000 X-ray radiographic images. Each image has a size of 2012 × 2012. These images were extracted from the patients files and are anonymized.

A selection of 600 images were studied and labeled by the experts in six classes (CVS1, …, CVS6). These labeled data were divided in three sets of Training, Validation and Testing. We did different division of the data: First, we had started by 300, 200 and 100, respectively, for Training, Validation and Testing. We did some preliminary tests with this small dataset. Then, we obtained more data, 600, then, 900 and, finally, about 1900. We used these images, step by step, in different experiences and used a Cross Validation (CV) technique by division and permutation of these data sets in different experiences.

**Figure 2:**
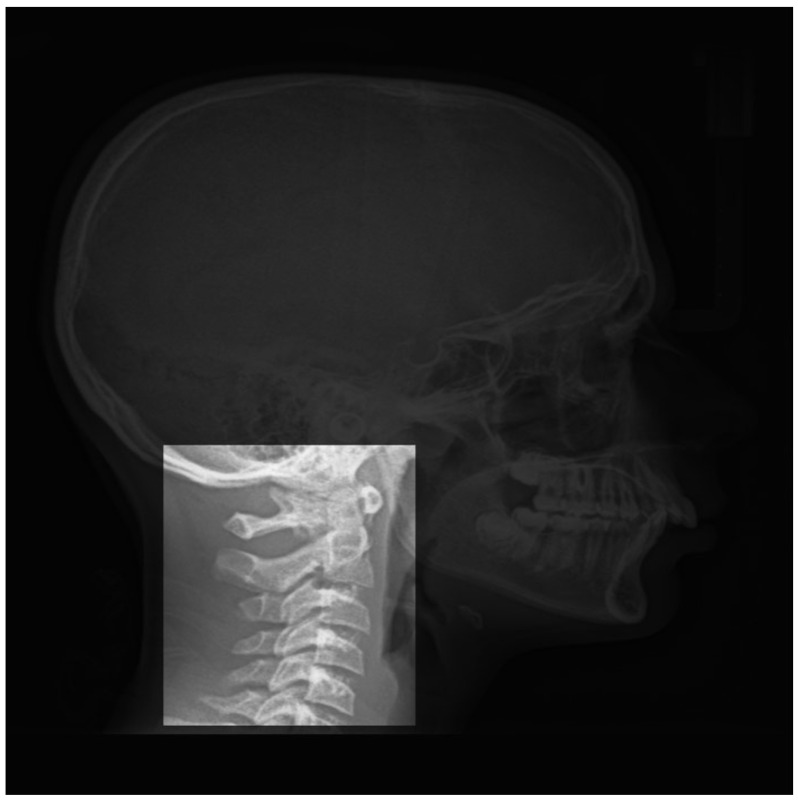
Isolated part (vertebra) of a standard radiography.

Also, as these images were from the whole head, only a specific part of the image was useful for this classification, we performed different preprocessing before feeding then to the DL input. Figure 2 shows one example of the interesting part of the images to be used for this application.

In a preprocessing step, each original image was first cropped to the interesting part (Test0: size 512 × 512), then resized to (Test1: size 256 × 256, Test2: 128 × 128 and Test3: size 64 × 64) or after resizing to 256 × 256, they are Sobel filtered to enhance the contours of the image (Test 4). These sets are used for studying the importance of the preprocessing the images.

**Figure 3:**
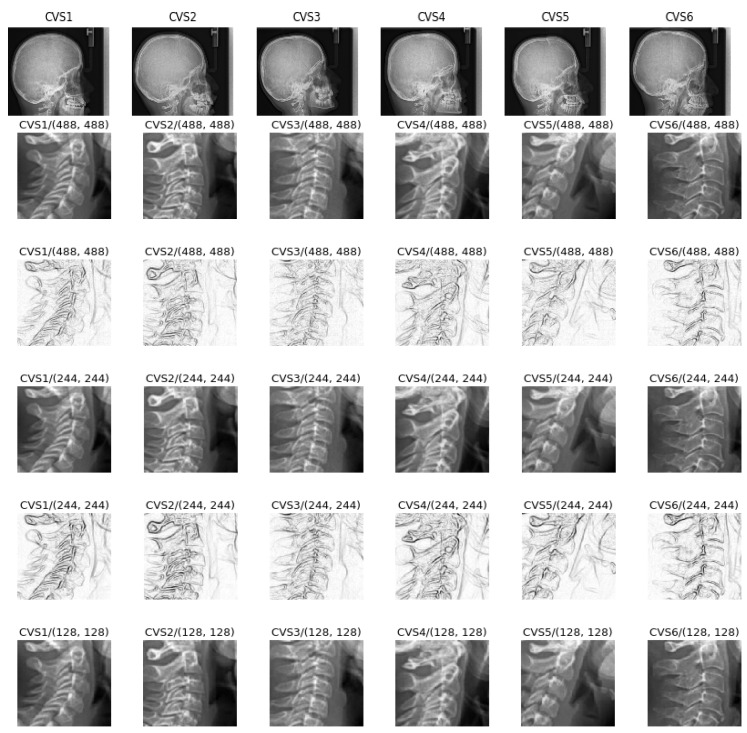
Originals and different preprocessing before training: (**a**) Originals (2012 × 2020), (**b**) test0: cropped images (488 × 488), (**c**) test1: cropped and sobel edge detector filter (488 × 488), (**d**) test2: cropped and resized (244 × 244), (**e**) test3: cropped, resized and sobel edge detector filter (244 × 244), (**f**) test4: cropped and resized (64 × 64).

We used other combinations of preprocessing with different cropping, resizing and filtering. Figure 3 shows in summary examples of these inputs.

### 4.2. Considered Deep Learning Networks

In a preliminary study, we used different Deep Learning network structures for this classification task. Several network architectures [30,31,32,33,34,35,36,37,38,39] have been proposed with success for ordinary photographic image classification. These different architectures have been proposed to deal with great problems of over fitting, vanishing or exploding gradients in the optimization parts of learning. Each one tries to provide a more efficient use of network parameters in order to offer ultimate improvement in the model efficiency in terms of computation time and accuracy.

The success that brought the breakthrough in the ImageNet competition in 2012 is related to the efficient use of Graphical Processing Units (GPU), data augmentation, rectified linear units, new dropout regularization and deep network architecture, where convolution operations were repeated multiple times between max-pooling operations. Since then, there has been a trend to make this style of network increasingly deeper through the use of small (3 × 3) convolutional filters such as VGG architecture [40,41,42,43,44,45,46,47,48].

However, very deep networks suffer from the problem of vanishing/exploding gradients during training process. To overcome this problem, He et al. [49] proposed residual networks termed ResNet by bypassing signal from one layer to the next via identity (or residual) connections. Recently, Huang et al. [50] took a step further by proposing dense networks (DenseNets) that connects each layer to every other layers in a feed-forward fashion. DenseNets offer several advantages, such as alleviating the vanishing gradient problem, encouraging feature reuse and substantially reducing the number of network parameters.

Almost all these networks have been designed and tested for ordinary color images on great data bases. Our main objective in this preliminary study was to compare these networks for our particular case of radiographic images.

We considered different classical networks:

- Resnet:
  Resnet was introduced in the paper “Deep Residual Learning for Image Recognition ” [49]. There are several variants with different output sizes, including Resnet18, Resnet34, Resnet50, Resnet101, and Resnet152, all of which are available from torchvision models. As our dataset is small, we used Resnet18 that we adapted in our case for 6 classes.
- Alexnet:
  Alexnet was introduced in the paper “ImageNet Classification with Deep Convolutional Neural Networks” [51] and was the first very successful CNN on the ImageNet dataset.
- VGG:
  VGG was introduced in the paper “Very Deep Convolutional Networks for Large-Scale Image Recognition” [52]. Torchvision offers eight versions of VGG with various lengths and some that have batch normalizations layers.
- Squeezenet:
  The Squeeznet architecture is described in the paper “SqueezeNet: AlexNet-level accuracy with 50× fewer parameters and <0.5 MB model size” [53]. It uses a different output structure than the other models mentioned here. Torchvision has two versions of Squeezenet. We used version 1.0.
- Densenet:
  Densenet was introduced in the paper “Densely Connected Convolutional Networks” [50]. Torchvision has four variants of Densenet. Here we used Densenet-121 and modified the output layer, which is a linear layer with 1024 input features and 6 classes, for our case.
- Inception v3:
  Inception v3 was first described in “Rethinking the Inception Architecture for Computer Vision” [54]. This network is unique because it has two output layers when training. The second output is known as an auxiliary output and is contained in the AuxLogits part of the network. The primary output is a linear layer at the end of the network. Note that when testing, we only consider the primary output.

### 4.3. Comparison of a Simple Network on Different Preprocessed Images

In a first step, to decide for a preprocessing module for our images, we designed a simple CNN network and tested its performances on different set of preprocessed images.

**Figure 4:**
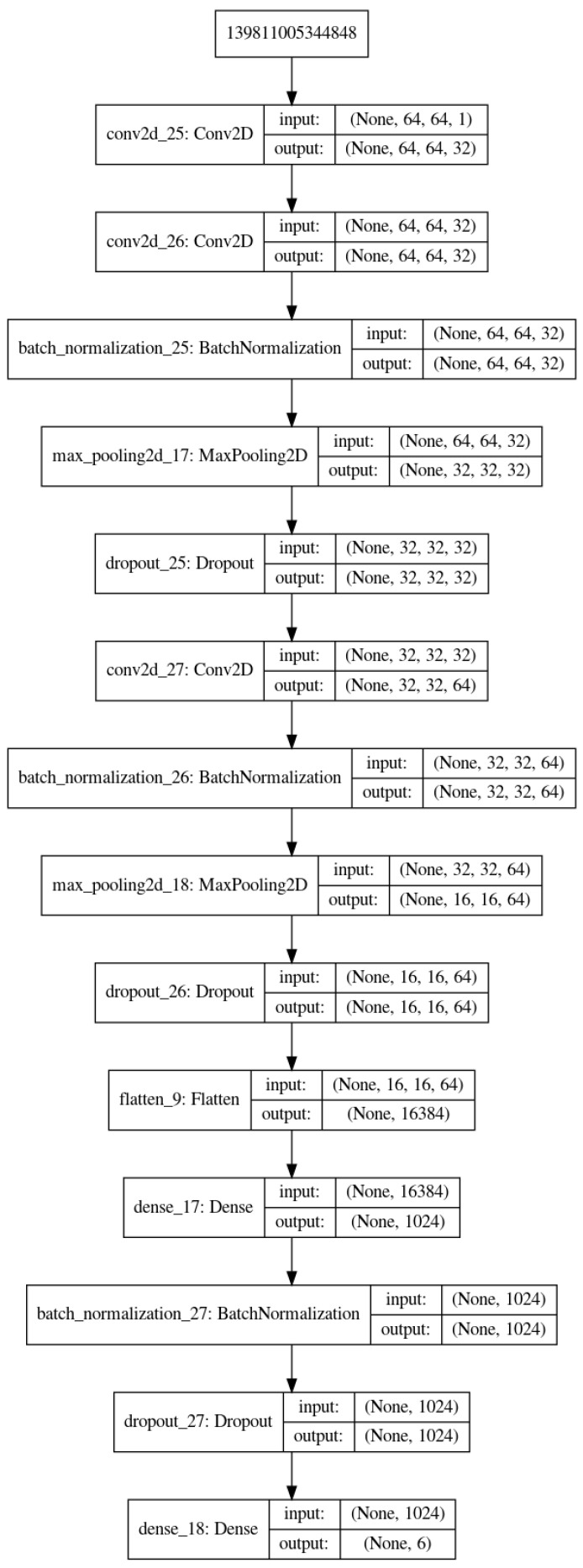
The structure of the proposed Deep Learning network for test with different preprocessed images.

As it can be seen on Figure 4, the structure of Deep Learning model is composed of an input convolutional layer and three or four other convolutional nets (CNN) layers and a fully connected of (32 × 32) to 6 classes. Each of the three CNNs is followed by a normalization, pooling and dropout layers with different dropout coefficients.

For this step, the models are trained with different partitions of the images in Training, Validation and Testing sets. The following figure shows 300 images which have been prepared for the training, then validated on 200 images and saved to be used for the testing step. A set of 120 images is used for testing and the average score was 80 percent.

### 4.4. Tools and Implementation

In this work, we used the following tools:

- SciKit-Image https://scikit-image.org/:
  All simple imag processing such as reading and writing images, Cropping, resizing and Filtering.
- SciKit-Learn https://scikit-learn.org/stable/: Data shuffling, Kmeans and Gaussian Mixture clustering, Principal Component Analysis and performance metrics.
- Keras wth tensorflow backend https://keras.io/:
  VGG16, VGG19 and ResNet50 convolution network models with ImageNet weights.
- PyTorch https://pytorch.org/:
  GPU and CPU vision algorithms, optimization, scheduler, feature transfer, etc.

We used all these tools for development of this project. As an example, we used Keras following this process.

- For a given Training data set, Create Model (keras.layers);
- Configure Model (model.compile);
- Train model (model.fit);
- For given Evaluating and Testing data, evaluate the trained model (loss = model.evaluate);
- Get prediction (pred = model.predict).

### 4.5. Prediction Results with Different Networks

With the implemented DL structure, we used 300 images for the training step, 200 images for the validation step and finally 150 images for the testing step. We had to fix a great number of parameters such as dropout rates, optimization algorithms, regularization parameters, etc.

**Figure 5:**
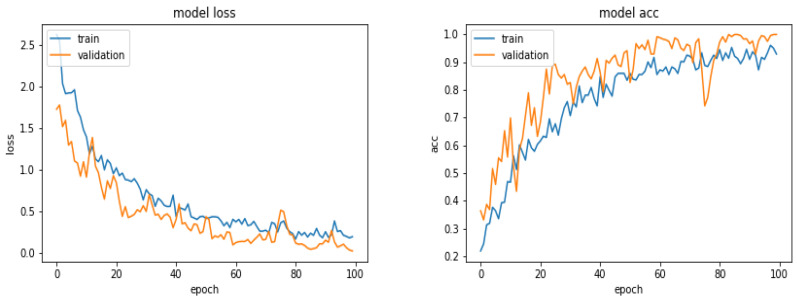
Evolution of the Loss function and the accuracy as a function of the epoch numbers.

Figure 5 shows the evolution of the Loss function and the accuracy as a function of the epoch numbers for one of these different tests.

Table 1 shows the prediction results obtained with different preprocessing of the data, both during the training and the testing

## 5. Proposed Method, Structure and Metrics

As we mentioned, ResNet, DenseNet, InceptionResNet and Xception are among the most efficient models that have achieved state-of-the-art accuracy on large image datasets such as CIFAR, ImageNet and all other classical photography images.

However, in our case, we have a small database. Hopefully, these images are not too different from each other and the variability (inter and intra classes) are not as large as in the case of photography images.

So, we decided to make our specific DL network, specifically adapted for our application and train it directly with our pre-processed images.

What we learned in previous sections can be summarized as follows:

- Almost all the pre-trained networks with feature transfer did not really work better. This is due to the fact that these networks are trained with great databases with photographic images which are more diverse than the radiographic images we have.
- A cropping of images of the size 512 × 512 to the specific informative part of cervical vertebra and possibly resizing them to 256 × 256 for reducing the computational costs are enough. However, to improve more, we tried different pre-processing of the images: mean, median and entropic filter to the images.
- The proposed method is the same as in Figure 3 with slightly different parameters which are optimized for our application.

### 5.1. Entropic Filtering

In classical information theory, information entropy is the log-base-2 of the number of possible outcomes for a message. In Bayesian probability theory, the entropy of a probability distribution $p=\{p_{1},\cdots ,p_{N}\}$ is defined as

(1) $$H\left(p\right)=-\sum_{n=1}^{N}p_{n}\ln p_{n}$$

From this definition, we see that, the highest entropy is obtained for a uniform distribution. We can use this property to measure the degree of uniformity of the distribution of the pixel values in an image. A local entropy of the distribution of a sliding window on an image can then be a measure of local uniformity of the image. Thus, for an image, local entropy is related to the complexity contained in a given neighborhood, typically defined by a structuring element. We use this property to pre-process the images as we are interested more on the contours than the gray levels of the images. Based on this, we can define an entropy filter which can detect subtle variations in the local gray level distribution.

**Figure 6:**
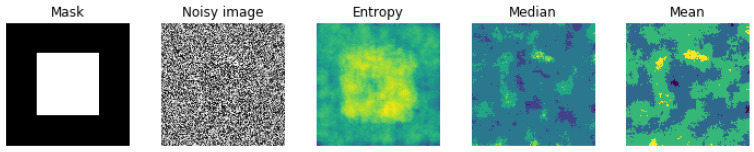
Comparison between three local filters: mean, median and entropy.

In image processing, there are many other local filterings, such as mean or median. To show the importance of our choice for the local entropy compared to the classical mean or median filtering, we show here the results of these filters on a noisy image obtained from a simple mask image with two constant value regions on which we added uniform noise. Figure 6 shows the results.

As it can be seen from Figure 6, the mean and the median filters are less effective than the entropy filter to catch the structural information of the image.

**Figure 7:**
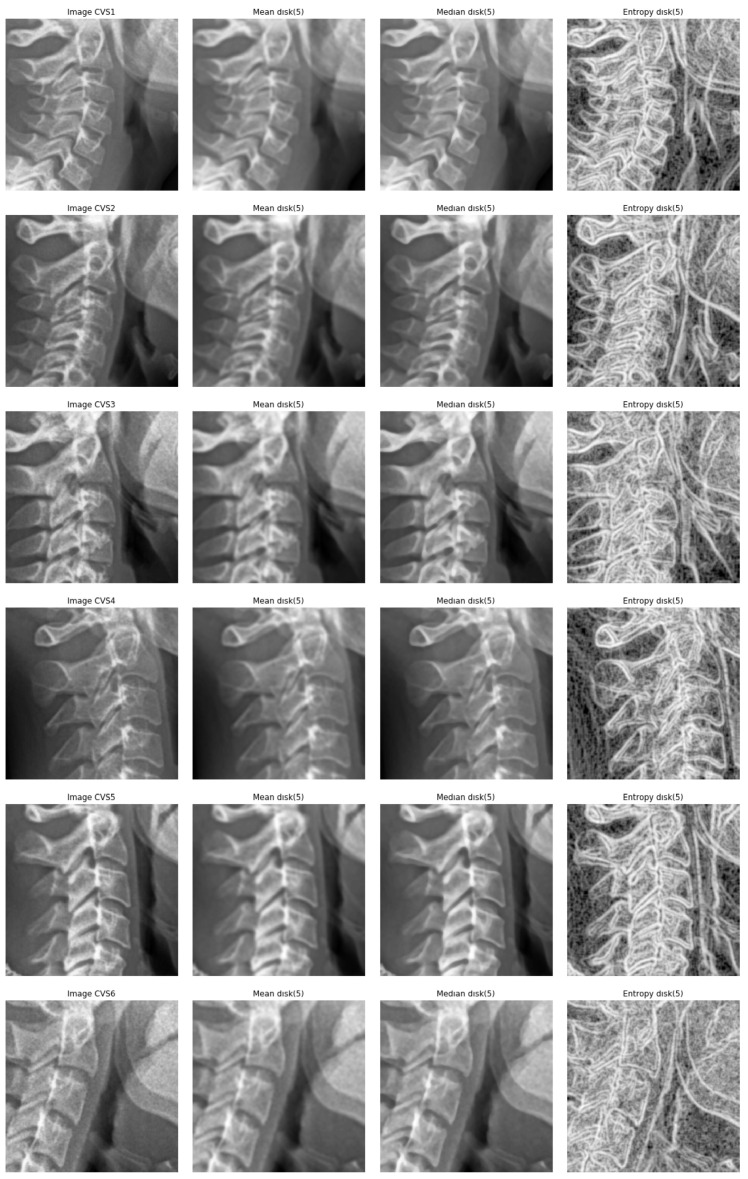
Comparison between three local filters: mean, median and entropy. Columns: from left to right: original, mean, median, entropy. Rows: from top to down CVS1, CVS2 to CVS6 cases.

Figure 7 show these filters on a sample of images on which we are interested in.

### 5.2. Optimisation Criteria

As we are interested in the general classification problem with six classes, we use the categorial cross entropy as the optimisation criteria for learning.

Categorical cross-entropy is a loss function that is used for single label categorization. This is when only one category is applicable for each data point.

Categorical cross-entropy will compare the distribution of the predictions (the activation in the output layer, one for each class) with the true distribution, where the probability of the true class is set to 1 and 0 for the other classes. If we note by $p_{ij}$ the probability that the sample *i* be in class *j* and by ${\hat{p}}_{ij}$ the probability of the predicted one, then cross-entropy is given by:

(2) $$H\left(p_{ij},{\hat{p}}_{ij}\right)=-\sum_{i}\sum_{j}p_{ij}\ln\frac{{\hat{p}}_{ij}}{p_{ij}}$$

When the true class is represented as a one-hot encoded vector, i.e., when the sample $x_{i}$ has the label $y_{ij}$ and the predicted label is noted ${\hat{y}}_{ij}$, the loss function associated to the cross-entropy becomes:

(3) $$L\left(y_{ij},{\hat{y}}_{ij}\right)=-\sum_{i}\sum_{j}y_{ij}\ln{\hat{y}}_{ij}$$

This is due to the fact that, $y_{ij}$ can take only the values 0 and 1. Thus, the closer the model’s outputs are to the training labels, the lower is this loss.

### 5.3. Optimisation Algorithms

We mainly used two standard algorithms: Stochastic Gradient Descent (SGD) and Adaptive Moment Estimation (Adam). We tried different variants of SGD (Batch and mini-batch without and with shuffling) with different learning rate and Adam with different learning rate and momentum. There is not really any easy way to fix these hyper parameters. We just tried different values to get the fastest convergence rate and a good generalization error. For a good review of these optimization algorithms, see [55] or [56].

### 5.4. Metrics Used to Measure the Quality of the Classification

Classical validation metrics used for two class classification (detection problem), such as True Positive (TP), False Positive (FP), True Negative (TN), False Negative (FN), Score or accuracy (S = TP/(TP + TN)), Precision (P = TP/(TP + FP)) and Recall (R = TP/(TP + FN)) are not enough for our multi class problem. The most complete metric is the confusion matrix.

### 5.5. Results

We proceeded many optimization of the parameters. For example, for the optimisations of the Learning Rate (LR) of the algorithm, we proceeded these steps:

- set a lower and an upper bounds for LR. For example: (1 × 10${}^{-1}$:1 × 10${}^{1}$);
- start the training of the network;
- increase the LR in an exponential way after each batch update;
- for each LR, record the loss at each batch update;
- train for a few epoches and plot the loss as a function of the LR;
- examine the plot and identify the optimal LR;
- update the LR and train the network for the full set of training data.
- apply the trained network to the evaluation and test data and look for a possibly over fitting problem;
- If all are right, then between those images in the evaluation and test set data, move those which are classified correctly with a very high probability to the training set and update the evaluation and test sets with new images until the end of the data base.

In the following, we show some partial results obtained with different sizes of the data base. In these experiments, we used 300 images as the validation and 300 as the test. In the following, we changed the number of images in the training set.

### 5.6. Results with 360 Images

**Figure 8:**
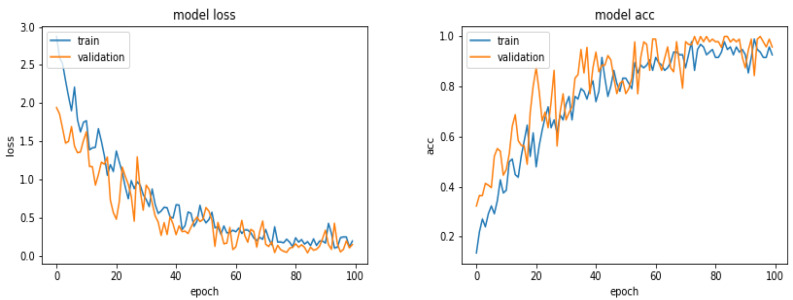
Evolution of the loss function and the accuracy as a function of the epoch numbers for the case of 360 images.

Figure 8 shows the loss and the accuracy functions during the learning and evaluation process when we used 360 images for training, 300 for evaluation and 300 for testing.

**Figure 9:**
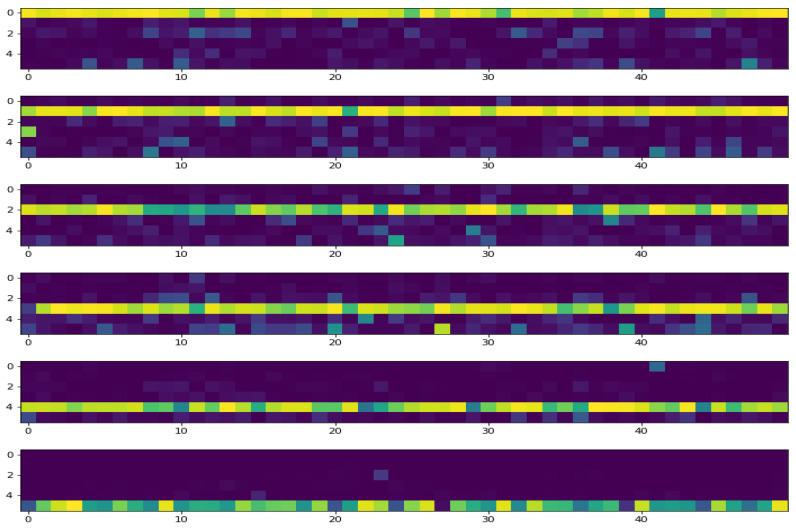
The prediction probabilities for each of the six classes are shown as an image. In each class, there are 50 images. The classes CVS1, …, CVS6 are shown from top to down.

To illustrate the accuracy of the test results, we show in Figure 9 the probabilities of the prediction as an image. As we have 50 images in each of the 6 classes, the prediction probabilities can be showed as an image of 6 lines and 50 columns for each class.

### 5.7. Results with 600 Images

**Figure 10:**
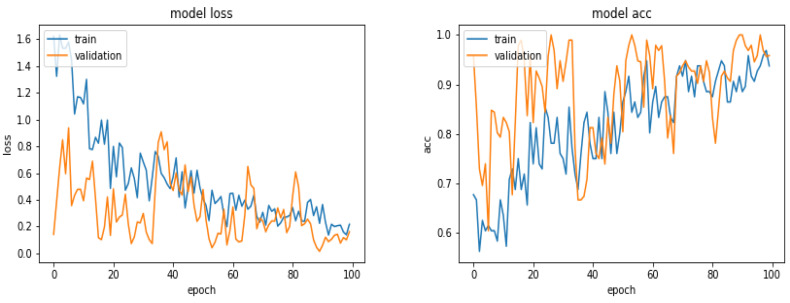
Evolution of the loss function and the accuracy as a function of the epoch numbers for the case of 600 images.

Figure 10 shows the loss and the accuracy functions during the learning and evaluation process when we used 600 images for training, 300 for evaluation and 300 for testing.

### 5.8. Results with 900 Images

**Figure 11:**
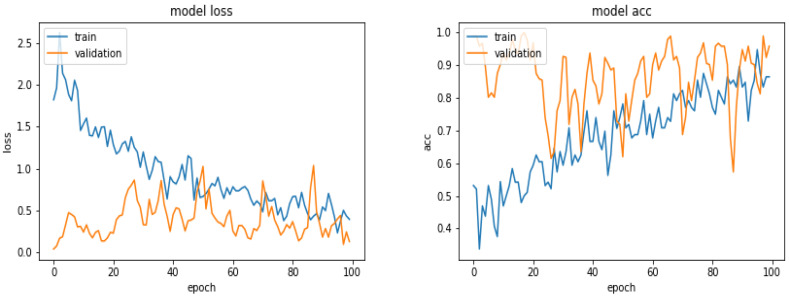
Evolution of the loss function and the accuracy as a function of the epoch numbers for the case of 900 images.

Figure 11 shows the loss and the accuracy functions during the learning and evaluation process when we used 900 images for training, 300 for evaluation and 300 for testing.

### 5.9. Results with 1870 Images

**Figure 12:**
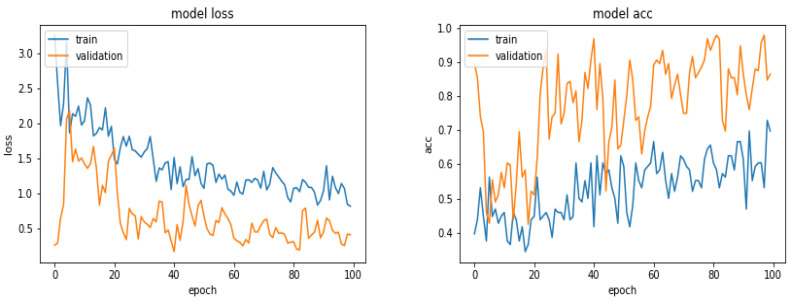
Evolution of the loss function and the accuracy as a function of the epoch numbers for the case of 1870 images.

Figure 12 shows the loss and the accuracy functions during the learning and evaluation process when we used all the available 1870 images for training, 300 for evaluation and 300 for testing.

### 5.10. Comparison of Accuracy for Different Number of Training Images

Table 2 shows the obtained accuracy on 300 test images when the models have been trained with different number of images: 360, 600, 900 and 1870.

As we can see from this table that, in general, when the number of training images increases, the global accuracy improves. However, the improvement is not linear. Also, when the numbers of images in each class are the same, a better accuracy is obtained. This can be seen in the table when the numbers of images increases from 360 to 900. In those cases, the number of images in each class was equally distributed. In the last case, where we used all the images, this equilibrium is not, since we have 199, 184, 825, 300, 200 and 162 images, respectively, in CVS1, CVS2, CVS3, CVS4, CVS5 and CVS6.

The results in this table show that the accuracy is increased when going from 360 to 600, but then decreased when going to 900 or even to 1870 images. This is probably due to the great diversity of the images and the fact that the number of images in each class is not the same. We are still exploring this database.

### 5.11. Comparison of the Results for Different Optimization Algorithms

We tried different optimization algorithms. Between all, here we report on two classical ones: SGD and ADAM.

**Figure 13:**
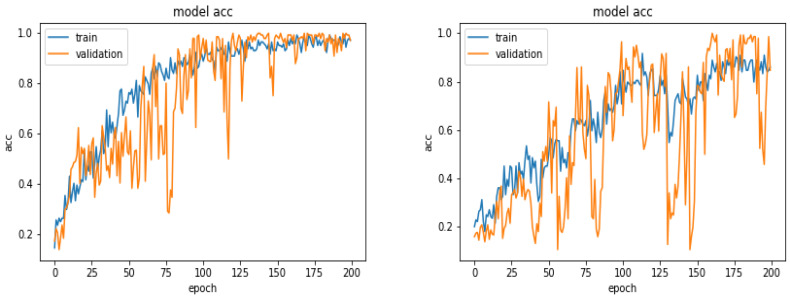
Evolution of the accuracy as a function of the epoch numbers for two different optimization algorithms SGD (**left**) and ADAM (**right**).

Figure 13 shows a comparison of the accuracy evolution during the optimization.

Table 3 shows a comparison of the accuracy obtained on the test data using Adam for different number of images during the training.

### 5.12. Comparison of the Results for Different Preprocessing

We tried different preprocessing of the images before starting the training. Here, we report only the case of entropic filtering which was the most significant.

**Figure 14:**
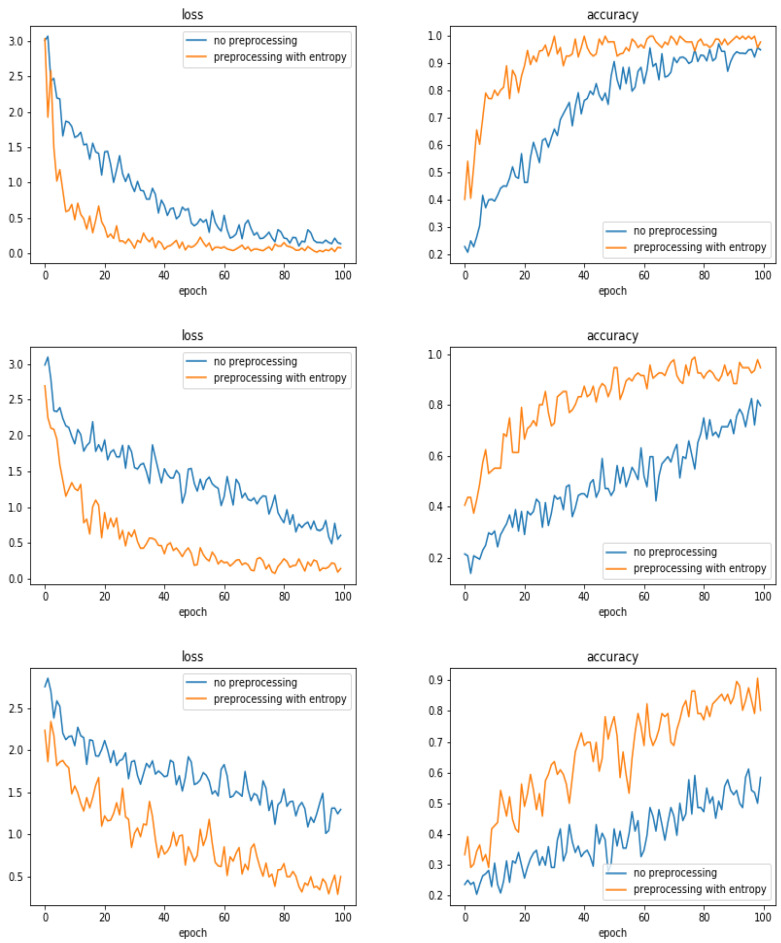
Evolution of loss and accuracy during the training as a function of the epoch numbers for two cases: without any filtering (**left**) and with entropic filtering (**right**). from top to botumn: The cases with 300, 600 and 900 training images.

Figure 14 shows the evolution of the accuracy as a function of the epoch numbers for the two cases of without any filtering and with entropic filtering.

Table 4 shows the summary of the obtained accuracy during the different tests.

### 5.13. Comparison of the Results as a Function of the Number of Layes

**Figure 15:**
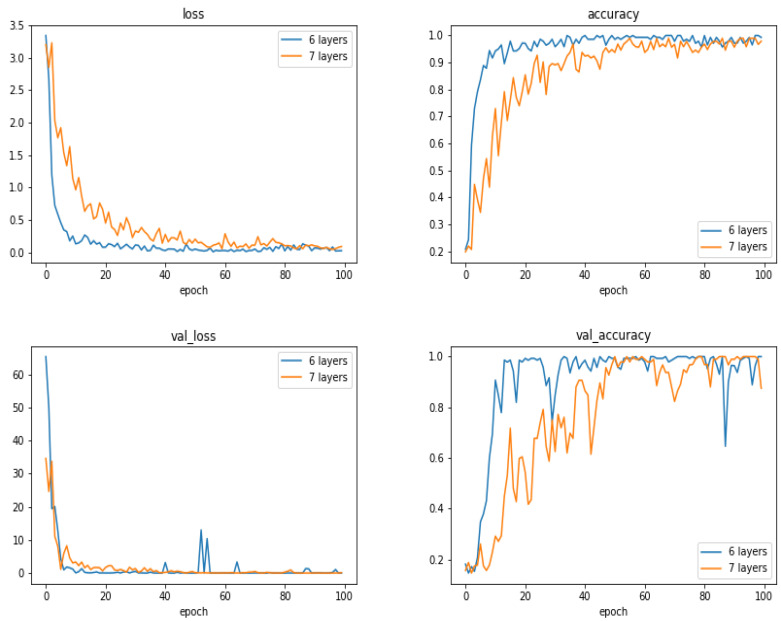
Evolution of loss and accuracy during the training (**upper row**) and during the validation (**lower row**) as a function of the epoch numbers for two cases: 6 layers and 7 layers networks. (The case with 300 images).

**Figure 16:**
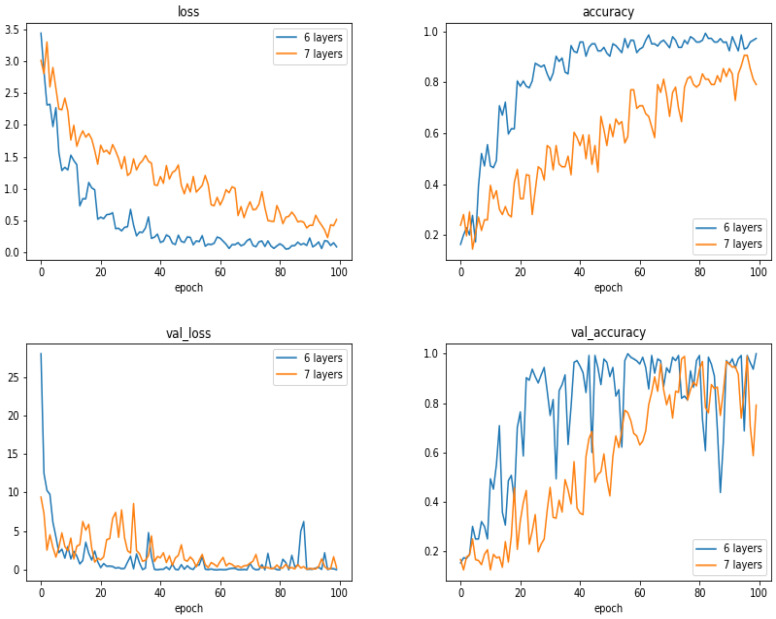
Evolution of loss and accuracy during the training (**upper row**) and during the validation (**lower row**) as a function of the epoch numbers for two cases: 6 layers and 7 layers networks. (The case with 900 images).

To see the performances of the proposed method as a function of the number of layers, we did a few experiments increasing the number of layers by one block of convolution, normalization, max-pooling and dropout layers. Figure 15 and Figure 16 and Table 5 show the comparison between the proposed 6 layers and 6 + 1 layer. As we can see, increasing the number of layers did not resulted to better performances.

In the Table 6, we show the confusion matrix and the classification report for the two last cases: 900 images for training, 300 for validation and 900 for testing.

### 5.14. Discussions

As we can see from the experiments and the results obtained, we may make a few remarks:

- Many existing CNN and DL structures have been developed particularly for photography images. They could not be used as they are for our X-ray radiography images. First, in general, they are constructed for colored images while the X-ray images are gray level. Using the pre-trained models cannot be used directly and even trying to fine tune them was not effective. Trying to train them directly was not successful, as in general, they need a very large number of images.
- We proposed a simple six-layer structure with five combination of convolution, normalization, max-pooling and dropout layers and one combination of dense, normalization and dropout layers. This structure was the most successful for our case.
- The choices of pre-processing, optimization criteria, optimization algorithms and optimization of hyper-parameters are very difficult. These need experience and deep knowledge of mathematics and techniques.
- We used categorical entropy as the optimization criteria, SGD or ADAM for optimization algorithm, and manually fixing the hyper-parameters using cross validation.
- Many performance criteria such as general accuracy, categorical accuracy, precision, recall and many other criteria, all can be derived from the confusion matrix and can be used to measure the performances of a classification method. In this paper, we mainly showed the accuracy.
- In our case, we show that when the training data are balanced, in general, the performances improve when the number of images increases. However, when the dataset is unbalanced, the situation is different.
- We did many experiments on the importance of the pre-processing of the images. Some pre-processings such as cropping on the interesting part of the image is almost always necessary, but other linear processing methods such as mean filtering or resizing did not have great impact. However, we discovered that an entropic filtering has improved the classification task.
- With small and moderate data, increasing the number layers does not improve the performances, and even, decrease them. A kind of balance between the number of parameters and the number of data has to be found. In our case, an approach of six compound layes (Convolution, Normalization, MaxPooling and DropOut) was the right one. When increased even one more layer, the performances decreased.

## 6. Conclusions

In this work, first, we described an image classification task which is important for the orthodontics community. More precisely, the objective was classifying the lateral radiographs of a great number of patients with the aim of determining the cervical vertebra maturation degree of bones, which is an important parameter for orthodontists. Then, we developed and presented a specifically designed classification method for this task.

In a first step, we tried different existing CNN and DL methods, models and algorithms to see if we can adapt and use them. The conclusion was that the existing models are, in general, for photography images. In our case, we had gray level X-ray radiography images. The adaptation of those methods could not be efficient. We then proposed a DL-CNN classification method which is particularly adapted for this task. The proposed model is a five-layer CNN with Batch Normalization, Max Pooling and Dropout layers. The optimization criteria is the Categorical Cross Entropy and the optimization algorithms we tested are SGD and Adam. To show the performances of the optimization algorithms during the training and evaluation, we used the training and validation loss and accuracy as function of number of epoches. Using the Confusion Matrix to compare the performances of the predictions, in this paper, we showed the obtained accuracy in each test.

To train the proposed model, in a first step, we used 360 first available labeled images for training, 300 for validation and hyper parameter tuning and finally 300 for testing. With these data, even if during the training and validation, we could obtain an accuracy of more than 95%, the accuracy for the testing images did not exceeded 90%. In a second step, we increased the number of the training dataset to 600, then to 900 and finally to all available 1870 images at the time of writing this paper. When going from 360 to 600 and 900 images equally distributed in the six classes, the performances improved and then stopped improving. For the case where we used the whole available 1870 images, the improvement was not really significant due to the fact that the numbers of images in the six classes were not equally distributed and there was greater diversity.

The dataset is still going to grow and we try to have equally distributed samples. During this development, we continue to develop other network structures and test other optimization algorithms. A clinically usable package is also planned within our project. This paper is an extended version of a communication at MaxEnt 2019 international workshop [57].

## Figures and Tables

**Table 1:** Comparison of the classification accuracy for different preprocessing.

Class	CVS1	CVS2	CVS3	CVS4	CVS5	CVS6
Test0	0.910	0.820	0.654	0.617	0.863	0.939
Test1	0.890	0.734	0.661	0.580	0.849	0.924
Test2	0.890	0.734	0.661	0.580	0.849	0.924
Test3	0.932	0.824	0.644	0.561	0.839	0.849
Test4	0.880	0.890	0.568	0.602	0.861	0.932

**Table 2:** Comparison of the classification accuracy for different number of training images. The optimization algorithm used for training was SGD.

No. of Images/Classes	CVS1	CVS2	CVS3	CVS4	CVS5	CVS6
360 images	0.917	0.977	0.970	0.980	0.870	0.850
600 images	1.000	0.997	1.000	0.993	1.000	0.997
900 images	0.980	0.890	0.993	0.963	0.933	0.927
1870 images	1.000	0.997	0.987	0.987	0.987	0.967

**Table 3:** Comparison of the classification accuracy for different number of training images. The optimization algorithm used for training was ADAM.

No. of Images / Classes	CVS1	CVS2	CVS3	CVS4	CVS5	CVS6
360 images	0.963	0.927	0.973	0.970	0.980	0.933
600 images	1.000	1.000	1.000	0.997	1.000	0.997
900 images	0.997	0.997	0.997	1.000	0.993	0.993
1870 images	0.990	0.970	0.953	0.980	0.977	0.990

**Table 4:** Effect of preprocessing: Comparison between no preprocessing and entropy filter preprocessing.

No. of Images/Classes	CVS1	CVS2	CVS3	CVS4	CVS5	CVS6
300 images:						
No preprocessing	1.000	0.983	0.987	0.997	0.923	0.990
Entropy filter	1.000	1.000	1.000	1.000	1.000	1.000
600 images:						
No preprocessing	0.978	0.975	0.952	0.973	0.937	0.925
Entropy filter	0.995	0.997	0.990	0.997	0.995	0.995
900 images:						
No preprocessing	0.794	0.826	0.635	0.833	0.783	0.817
Entropy filter	0.984	0.984	0.988	0.996	0.993	0.996
1870 images:						
No preprocessing	0.879	0.880	0.651	0.833	0.885	0.911
Entropy filter	0.882	0.857	0.637	0.852	0.897	0.919

**Table 5:** Effect of the number of layers: Comparison between two cases of 6 and 7 layers. Increasing the number of layers decreases the score of the classification.

No. of Layers/Classes	CVS1	CVS2	CVS3	CVS4	CVS5	CVS6
300 images:						
6 layers:	0.913	0.850	0.802	0.912	0.895	0.913
7 layers:	0.892	0.805	0.890	0.793	0.885	0.897
900 images:						
6 layers:	0.998	0.998	1.000	0.988	0.994	0.991
7 layers:	0.930	0.939	0.952	0.924	0.966	0.969

**Table 6:** Confusion matrix and classification report for the two cases of 6 layers and 7 layers.

Case with 6 Layers				Case with 7 Layers			
Confusion Matrix				Confusion Matrix			
[[101 6 4 25 12 2]				[[101 6 4 25 12 2]			
[ 0 110 3 30 5 2]				[ 0 110 3 30 5 2]			
[ 0 0 122 22 6 0]				[ 0 0 122 22 6 0]			
[ 0 0 0 148 2 0]				[ 0 0 0 148 2 0]			
[ 1 1 1 8 139 0]				[ 1 1 1 8 139 0]			
[ 0 0 0 16 2 132]]				[ 0 0 0 16 2 132]]			
Classification Report				Classification Report			
precision	recall	f1-score	support	precision	recall	f1-score	support
CVS1 0.99	0.67	0.80	150	CVS1 0.99	0.67	0.80	150
CVS2 0.94	0.73	0.82	150	CVS2 0.94	0.73	0.82	150
CVS3 0.94	0.81	0.87	150	CVS3 0.94	0.81	0.87	150
CVS4 0.59	0.99	0.74	150	CVS4 0.59	0.99	0.74	150
CVS5 0.84	0.93	0.88	150	CVS5 0.84	0.93	0.88	150
CVS6 0.97	0.88	0.92	150	CVS6 0.97	0.88	0.92	150
